# New Uses for Mica

**Published:** 1869-06

**Authors:** 


New Uses for Mica.—Few uses to which mica can be placed have been found up to the present time. M. Puscher lately drew the attention of the Industrial Society of Nuremberg, to the Siberian mica, which occurs in very fine plates, and indicated some new purposes to which it could be applied. When the thin plates of mica are cleaned with concentrated sulphuric acid, and silvered in the same way as glass, they take a lustre similar to that of silver, and being pliable they can be employed in the covering of various ornaments. By heating the thin plates and afterwards exposing them for a very short time in a muffle heated to bright redness, an aspect of matted silver is given. It is necessary to avoid heating the mica too long or too powerfully, since in either case a yellow shade is communicated, as well as great brittleness. The silvery substance formed is distinguished from metals by the property of resisting nearly all re-agents; it is not in the least altered by sulphuretted combinations, by the sun, water, air, concentrated acids or alkalies.—*Chemical News*.

